# SeqAnt: A web service to rapidly identify and annotate DNA sequence variations

**DOI:** 10.1186/1471-2105-11-471

**Published:** 2010-09-20

**Authors:** Amol Carl Shetty, Prashanth Athri, Kajari Mondal, Vanessa L Horner, Karyn Meltz Steinberg, Viren Patel, Tamara Caspary, David J Cutler, Michael E Zwick

**Affiliations:** 1 Department of Human Genetics, Emory University School of Medicine, Atlanta, GA, USA; 2 Graduate Program in Population Biology, Ecology and Evolution, Emory University, Atlanta, GA, USA

## Abstract

**Background:**

The enormous throughput and low cost of second-generation sequencing platforms now allow research and clinical geneticists to routinely perform single experiments that identify tens of thousands to millions of variant sites. Existing methods to annotate variant sites using information from publicly available databases via web browsers are too slow to be useful for the large sequencing datasets being routinely generated by geneticists. Because sequence annotation of variant sites is required before functional characterization can proceed, the lack of a high-throughput pipeline to efficiently annotate variant sites can act as a significant bottleneck in genetics research.

**Results:**

SeqAnt (*Seq*uence *An*notator) is an open source web service and software package that rapidly annotates DNA sequence variants and identifies recessive or compound heterozygous loci in human, mouse, fly, and worm genome sequencing experiments. Variants are characterized with respect to their functional type, frequency, and evolutionary conservation. Annotated variants can be viewed on a web browser, downloaded in a tab-delimited text file, or directly uploaded in a BED format to the UCSC genome browser. To demonstrate the speed of SeqAnt, we annotated a series of publicly available datasets that ranged in size from 37 to 3,439,107 variant sites. The total time to completely annotate these data completely ranged from 0.17 seconds to 28 minutes 49.8 seconds.

**Conclusion:**

SeqAnt is an open source web service and software package that overcomes a critical bottleneck facing research and clinical geneticists using second-generation sequencing platforms. SeqAnt will prove especially useful for those investigators who lack dedicated bioinformatics personnel or infrastructure in their laboratories.

## Background

Next generation sequencing platforms are making the often expensive and slow process of detecting DNA sequence variation a relic of the past. Today, individual investigators are able to harness enormous raw sequencing power at a dramatically lower cost per sequenced base[1,2]. Adding to the utility of these second-generation of sequencing platforms, recent development and validation efforts have explored better methods of isolating target DNA from complex eukaryotic genomes[3-10], see review in[11]. Applying these methods to perform targeted sequencing of human exomes has shown that we can indeed identify causative variants underlying monogenic disorders[12,13]. The use of targeted or genome-wide sequencing in forward genetic screens of model organisms is likely to become increasingly used to identify causative mutations. In the field of human genetics, although sequencing entire human genomes for complex disease studies may still be expensive, the number of human genomes sequenced is ever-increasing, while the cost of sequencing a human genome continues to fall[14-22]. Furthermore, there is a growing appreciation for the role of rare genetic variants in the etiology of complex human diseases[23-25]. Importantly, this particular class of variation can only be discovered through direct sequencing. Clearly, the future of clinical and research genetics is tied to the efficient determination of genome sequences.

Despite their promise, application of these technologies to targeted or whole-genome sequencing faces a number of immediate bioinformatic challenges, especially for investigators without access to bioinformatics expertise. Each genome-wide sequencing experiment is expected to identify tens of thousands to millions of variant sites. Many of these variants will be rare and previously undiscovered. The ultimate goal is to identify the single causative variant, or a subset of functional variants, that may either obviously explain a given phenotype, or may warrant further characterization in future functional studies. An individual geneticist using next generation sequencing technologies is faced with the challenge of using information from multiple publicly available databases in attempting to rapidly annotate this vast number of DNA sequence variants. The previous and widely used method, whereby an individual researcher annotates variant sites by querying a database like the UCSC Genome Browser to obtain the relevant information, is not only tedious, but poses a major bottleneck in interpreting genome sequences for the larger studies that individual investigators are now pursuing[26]. Other software packages focus mainly on providing annotation for limited genomic regions relevant to genome-wide association studies in humans[27-29]. The most comparable website available, the GVS: Genome Variation Server, is focused solely on annotating human and mouse genomic variation, while excluding the fly and worm genomes[30]. Furthermore, the underlying software is neither open source nor freely available, which prevents a user from establishing a local mirror site. While individual laboratories might choose to write their own software to automate this process, it would be far more efficient for genetics researchers to be able to access a web service capable of solving this problem, thereby allowing them to focus their efforts on downstream experiments.

To address these issues, we have developed an open source web-based software package, named SeqAnt (Sequence Annotator), which automates the process of DNA sequence annotation. Individual investigators are able to quickly obtain detailed information for those variants identified in their experiments, either using the SeqAnt website or by obtaining the source code from the authors and processing batch files locally. Here we demonstrate the performance of SeqAnt on a variety of mammalian datasets characteristic of the types of applications next-generation sequencing can bring to the study of complex eukaryotic genomes.

## Implementation

### The design and implementation of SeqAnt

Sequence data requiring annotation can be uploaded and results viewed using an interactive, web-based graphical user interface (GUI) that has been tested for standard browsers. Data can be uploaded to SeqAnt in four main commonly used data formats that can be easily generated by genetics researchers:

1. A set of three files, which include a reference fasta sequence file, a multifasta sequence file, and a genome position file in a Browser Extensible Data (BED) file format that maps the individual sequence file fragments against the appropriate reference genome.

2. A list-of-variations file that includes the chromosome and fragment position, the relative position in a sequenced fragment, the reference allele, the minor allele and the type of variation (SNP or Indel).

3. A list of variations similar to that in (2) above with additional information that includes the Sample ID for each individual within a given experimental study.

4. A single variant with its chromosome, its genomic position, and its variant type.

The SeqAnt source code and associated database can be downloaded for batch processing on a user's local computer[31]. The SeqAnt website contains a detailed help section, example files, and a video tutorial demonstrating how to use the various options on the site[32].

Annotated output data can be displayed in a variety of ways. The GUI provides a simple and convenient interface for the user to select and highlight the annotation information associated with a given variant or to view by functional class. The user may also download the annotated variation in a tab delimited text file format or in a BED format that can be viewed in the UCSC Genome Browser. The specific annotation field outputs are found in Table 1.

**Table 1:** Annotation information output by SeqAnt.

Field ID	Annotation Field	Description
1	Chromosome	Chromosome containing variant site

2	Genome Position	Absolute position of variant site on a chromosome

3	Gene Name	Name of the locus containing the variant site*

4	Gene Strand	Orientation of locus*

5	Functional Category	Annotated functional category for variant site

6	Reference Allele	Reference allele at the variant site

7	Minor Allele	Minor allele at the variant site

8	Variation Type	Type of variant (either SNP or Indel)

9	Reference Amino Acid	Reference amino acid at variant site**

10	Amino Acid Position	Position of the amino acid in the peptide chain**

11	Modified Amino Acid	Modified amino acid due to variant site**

12	Warnings	Possible errors (if any) detected in the RefSeq annotation

13	RefSeq ID	RefSeq ID reported by the UCSC track

14	dbSNP ID	dbSNP ID if the variant site has already been reported

15	dbSNP Heterozygosity	Corresponding dbSNP heterozygosity if variant site has already been reported

16	dbSNP Orientation	Corresponding dbSNP orientation of variant site if it has already been reported

17	PhastCons Score	PhastCons score for variant site

18	Sample IDs	List of sample IDs with variant (when multiple sample IDs are present)

Some annotation fields only apply to specific functional categories or input data types (* Exonic, UTR; ** Exonic)

The SeqAnt web-based GUI has been scripted mainly in PHP: Hypertext Preprocessor (PHP) language using HyperText Markup Language (HTML) and Javascript to enable user-interaction. The web-based GUI allows the user to upload the required files for annotation and in turn invokes the back-end scripts that query the SeqAnt database to annotate the variations and tabulate the results. The back-end scripts are written in Perl and are available for download allowing the user to execute the command-line version of SeqAnt on a local machine. The results obtained from the back-end Perl scripts are displayed on the web-based GUI and are navigable by the user. These results can also be downloaded to the user's local machine for future reference. Whereas the command line version is more useful for automated batch processing of user data, the web interface is more appropriate for user interactivity. Along with the annotation result files, a log file is delivered that contains technical details involving the execution of the annotation process and any discrepancies that may have been found in the original uploaded data.

### External resources used by SeqAnt

The SeqAnt database consists of a series of binary files generated by preprocessing a set of external database resources obtained from the UCSC Genome Browser[26]. Rapid queries of the custom binary database aid in the quick delivery of annotation results. The specific public databases used to create the preprocessed binary files include:

#### 1. UCSC Genome Assembly Track

The UCSC Genome Assembly track contains the assembled genomic DNA sequences in fasta format. Presently, SeqAnt is able to annotate variants relative to the following genomes: hg19 human assembly released in February 2009, hg18 human assembly released in March 2006, the mm9 mouse genome assembly released July 2007, the BDGP-R5/dm3 *Drosophila melanogaster *genome assembly released in April 2006, and the WS190/ce6 *Caenorhabditis elegans *genome assembly released in May 2008.

#### 2. UCSC RefSeq Gene Track

The RefSeq Gene track provides information about the genes belonging to a particular genome for a specific build in a tabulated format. The information includes the chromosomal location of each individual gene, its transcriptional region, the locations of each of its exons that would be translated to form a protein, gene names, RefSeq accession numbers, and exon frames, all of which are processed into positional information and stored into a binary file for quick access. SeqAnt uses the corresponding RefSeq tables for the hg19, hg18, mm9, dm3 and ce6 builds.

#### 3. UCSC SNPs Track

The variation track contains a listing of known variant sites. For the hg19 and hg18 assemblies, the latest dbSNP build 130 has been used. This build includes variants from the 1000 Genomes project. For the mouse mm9 assembly, SeqAnt uses the dbSNP build 128. There are no SNP tracks for the dm3 and cd6 assemblies.

#### 4. UCSC Conservation Track

The UCSC Conservation track holds the information for the phastCons conservation scores in wiggle file formats[33,34]. For the human hg19 build, SeqAnt uses the latest Conservation Track released in October 2009, which includes from the multiple sequence alignment of 45 vertebrate species to the human genome (including subsets of nine primates and 32 placental mammals). For the human hg18 build, SeqAnt uses the latest Conservation Track released in January 2009, which includes from the multiple sequence alignment of 43 vertebrate species to the human genome (including subsets of eight primates and 31 placental mammals). Similarly for the mouse mm9 build, the *D. melanogaster *dm3 build, and the *C. elegans ce6 *build, SeqAnt uses the latest Conservation Tracks consisting of 30 vertebrate species, 14 insect species, and five nematode species respectively.

For the field IDs listed in Table 1, fields (1), (2), (6), (7), (8) and (18) are extracted from the input data provided by the user. Fields (3), (4), (5), (9), (10), (12) and (13) are obtained from the UCSC RefSeq Gene track. (11) is determined on the basis of the variant site. (14), (15) and (16) are obtained from the UCSC SNPs track while (17) is derived from the UCSC Conservation track. The data from the above external resources have been downloaded and preprocessed to generate the binary database used by SeqAnt in order to annotate the input data[31,32]. The binary database aids in the quick delivery of the annotation results.

### Recessive and compound heterozygote detection

SeqAnt provides the user with a listing of variants at replacement sites that are either recessive or compound heterozygotes within each individual analyzed. A summary file provides a complete listing of genes containing these variants. Three criteria have to be met for recessive and compound heterozygote detection. First, one or more homozygous positions or two or more heterozygous replacement variants are required to fall within the same locus. Second, none of the variant positions should have been reported previously in dbSNP. Third, the phastCons score for each variant position should be above a user-defined threshold.

## Results and Discussion

### Datasets analyzed with SeqAnt

A total of six sequence datasets of various sizes and types were analyzed by SeqAnt (Table 2). In order to perform the time comparison, all datasets were provided to SeqAnt in the "List of Variants" format. The first consisted of the sequence of a 48-kb region, including the FMR1 locus[4]. The second consisted of the microarray-based genomic selection (MGS) of 390 kb from a larger 700-kb sized region in a mouse containing the *hennin *mutation, which is known to consist of a variant site in a splice donor site in the Arl13b locus[4,9,35]. Mouse analysis was performed in accordance with approved protocol from the Emory IACUC. The third consisted of MGS targeted sequencing of a 329-kb region in ten HapMap samples[9]. The fourth consisted of eight recently published whole-exome sequence datasets obtained from the NCBI Sequence Read Archive[12]. These were mapped to the UCSC hg18 build and the bases called with the Emory Mapper (Cutler and Zwick, unpublished data). Variants detected were annotated with SeqAnt. The fifth genome was sequenced by the ABI 3730 sequencer followed by the base-calling which was done with KB v1.2. The internal HuRef variants were downloaded and formatted into a variations list file that could be used with SeqAnt to annotate the variant sites[16]. The sixth dataset was the genome sequence of a Korean male sequenced and analyzed using the Illumina Genome Analyzer paired-end sequencing and software[19]. The dataset consisted of a list of SNPs. All SeqAnt results from these analyses are available from the corresponding author upon request.

**Table 2:** SeqAnt sequence annotation total sites and execution time.

	Okou et al. 2007	Caspary et al. 2007	Okou et al. 2009	Ng et al. 2010	Levy et al. 2007	Kim et al. 2009
Genome	Human	Mouse	Human	Human	Human	Human

Size of Region Sequenced (kb)	48	683	329	~26,000	~3,000,000	~3,000,000

Individuals Sequenced	1	1	10	8	1	1

Total Variant Sites Annotated	37	1375	13,739	61,451	3,296,384	3,439,107

Execution Time	0.17s	0.72s	4.58s	27.28s	28 m 45.3s	28 m 49.8s

Exonic Replacement SNPs	1	2	28	31,154	8,955	9,746

Exonic Silent SNPs	1	0	9	30,233	9,692	10,818

Exonic Indel Sites	0	9	243	0	365	0

UTR SNPs	4	2	91	1,931	26,751	30,798

UTR Indels	0	2	481	0	3,238	0

Intronic SNPs	21	46	1,347	70	1,109,359	1,246,439

Intronic Indel Sites	0	309	5,803	0	139,192	0

Intergenic SNPs	10	289	1060	18	1,921,438	2,141,948

Intergenic Indel Sites	0	716	4683	0	205,709	0

### Results of SeqAnt analysis

**Figure 1:**
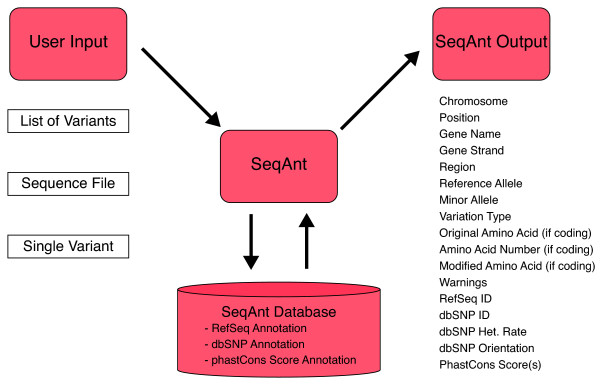
**Overview of SeqAnt**. SeqAnt accepts three main types of user input, obtains annotation information from the SeqAnt Database, and returns the detailed annotation output to the user.

Sequence data requiring annotation for either the human or mouse genomes are uploaded to the SeqAnt website in three possible formats: a multi-fasta file with associated genomic positions, a list of variants and positions, or simply the position of a single genomic location. The SeqAnt program then determines the annotation information for the variable sites (Table 1). The resulting output can be viewed on a web-based graphical user interface that is tested for standard browsers, in a series of downloadable tab-delimited text files, or in a downloadable BED file format that can be imported into the UCSC browser (Figure 1).

**Figure 2:**
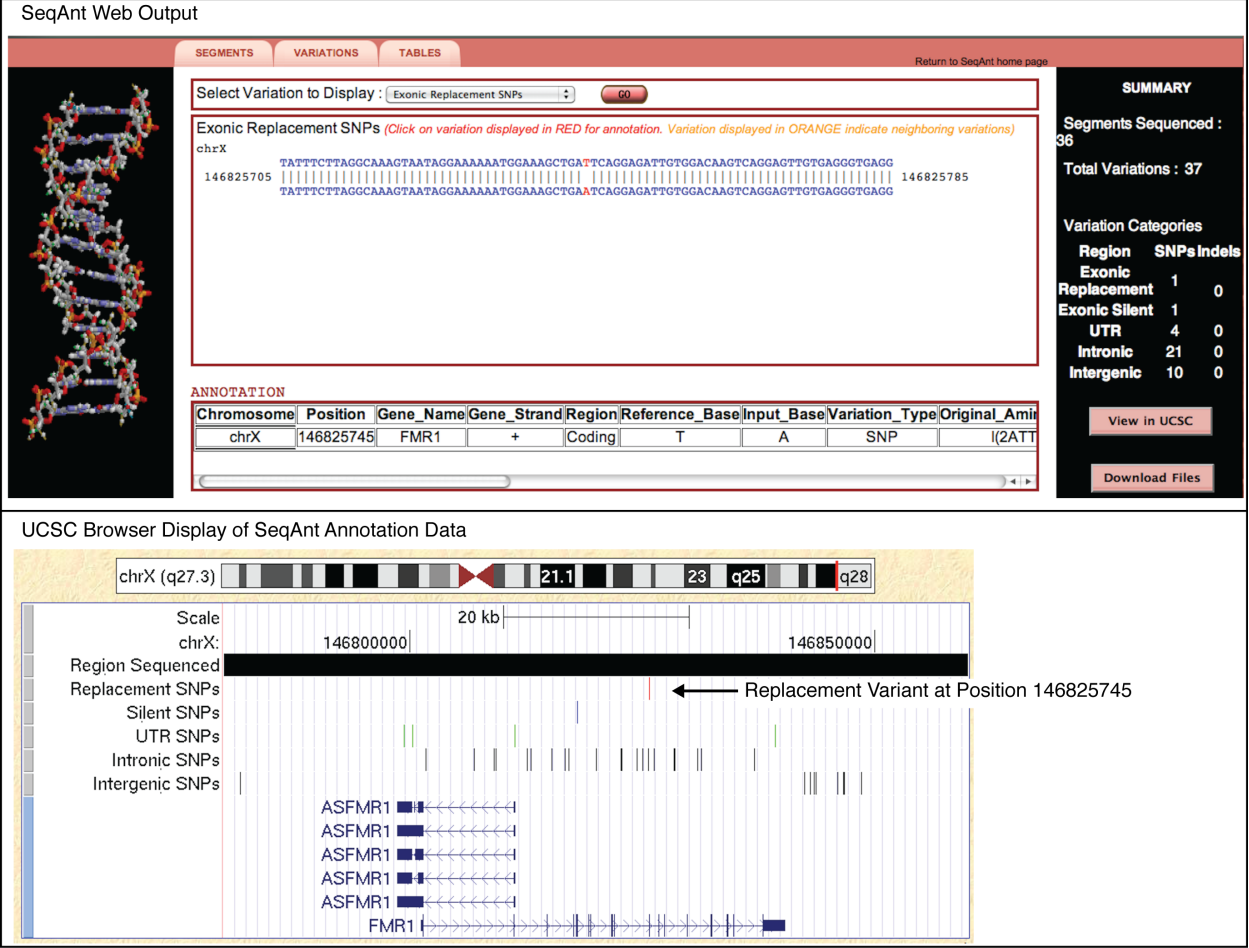
**Examples of SeqAnt Output**. The upper panel shows the SeqAnt web output for the I403N FMR1 mutation. The lower panel shows the UCSC view of the BED format SeqAnt output file.

As a direct demonstration of the utility of SeqAnt for detecting functional variants, we first annotated the data from a 48 kb resequencing experiment of a single human sample with a known coding sequence mutation at the FMR1 locus[4]. A total of 37 variant sites were identified and annotated in 0.17 seconds, including the I304N mutation, which has been shown to result in intellectual disability[36] (Table 2). Figure 2 shows the SeqAnt web output for the I304N variant site, in addition to the UCSC browser output of all the variant sites (Figure 2).

SeqAnt can also be used to annotate variants from other eukaryotic genomes, including the mouse, *D. melanogaster*, and *C. elegans*. As an example of its potential in mouse genetics, we performed the targeted resequencing of 390 kb from a larger 700-kb sized region containing the *hennin *mutation, which is known to consist of a variant site in a splice donor site in the Arl13b locus[35]; a total of 1375 variant sites were identified and annotated in 0.72 seconds (Table 2). SeqAnt identified the known recessive *hennin *allele in the heterozygous state and reported it as being a possible splice donor variant site.

To assess the speed of SeqAnt, we next annotated a series of publicly available datasets that ranged in size from 13,739 to 3,439,107 variant sites (Table 2). We performed sequence mapping and assembly of the eight recently published HapMap exomes[12], identifying a total of 61,451 variant sites. SeqAnt was able to annotate these sites from the eight exomes in 27.28 seconds, thereby showing clearly that SeqAnt overcomes a significant data analysis bottleneck facing researchers using exome sequencing protocols. Finally, annotating the more than 3 million variant sites from an entire human genome took less than 30 minutes per genome, making SeqAnt a valuable tool for even the largest sequence-based studies of complex eukaryotic genomes.

SeqAnt can also be used to rapidly detect those loci in a given sample harboring either two identical or two different recessive replacement alleles that are not found in dbSNP. This feature is particularly useful when analyzing exome sequencing datasets, since it allows a researcher to immediately identify the list of loci harboring alleles consistent with recessive inheritance of a given phenotype. For example, in the eight exomes sequence by[12], SeqAnt reported that the total number of loci meeting this criterion ranged between 370 (NA19240) and 567 (NA12156). These results are easily obtained for each sample analyzed with SeqAnt in the downloaded summary files and can help users rapidly identify loci harboring recessive alleles that may account for an observed phenotype.

## Conclusions

High-throughput, inexpensive DNA sequencing is radically transforming the field of genetics. Sequence-based studies of exomes or complete complex eukaryotic genomes are expected to uncover a large number of DNA sequence variants. Though many variants may have been observed already, some will be novel. Rapid, comprehensive DNA sequence annotation of variants is a critical step that must be performed before a genetics researcher can prioritize the further functional characterization of variants discovered by genome sequencing.

SeqAnt overcomes this bottleneck by allowing investigators or core sequencing facilities to rapidly annotate their sequence data via either a web service, or by downloading and locally running an open source implementation. SeqAnt is highly efficient and easily scales to whole human exome and genome sequencing. The SeqAnt website currently supports the human, mouse, fly and worm genomes. Additional genomes of interest will be added in the future. Furthermore, the annotation data reported by SeqAnt can be expanded easily as this information becomes available and can also be readily tailored for specific applications. For example, integrating SeqAnt into a human clinical DNA sequencing pipeline is straightforward. Ultimately, we believe that SeqAnt means individual investigators can now have some of the capabilities typically found only at a traditional genome sequencing center, without requiring that they independently develop the software. SeqAnt overcomes a significant bottleneck that can slow the wide-scale application of next-generation sequencing for a host of human and model organism genetics research and human clinical genetics applications.

## Availability and Requirements

Project name: SeqAnt (*Seq*uence *Ann*otator)

Project home page: http://seqant.genetics.emory.edu, http://seqant.sourceforge.net

Operating system(s): Platform independent

Programming language: Perl, PHP, HTML, Javascript

Other requirements: None

License: GNU GPL

Any restrictions to use by non-academics: SeqAnt is provided free for use in accordance with the GNU GPL license.

## List of Abbreviations

SeqAnt: Sequence Annotator; GUI: graphical user interface; BED: Browser Extensible Data; MGS: microarray-based genomic selection; PHP: Hypertext Preprocessor; HTML: HyperText Markup Language.

## Authors' contributions

ACS, TC, DJC and MEZ conceived the project and wrote the paper. ACS, PA, VP, DJC, MEZ designed the software and performed the research. ACS implemented the software, built the databases, and designed the web-based interface. KM, VLH, and KMS performed the targeted sequencing experiments. All authors read and approved the final manuscript.
